# Methylthioadenosine phosphorylase (MTAP)-deficient T-cell ALL xenografts are sensitive to pralatrexate and 6-thioguanine alone and in combination

**DOI:** 10.1007/s00280-015-2747-2

**Published:** 2015-04-28

**Authors:** Philip M. Tedeschi, Yamini K. Kathari, Nadine Johnson-Farley, Joseph R. Bertino

**Affiliations:** Departments of Pharmacology and Medicine, Rutgers Cancer Institute of New Jersey, Rutgers, The State University of New Jersey, 195 Little Albany Street, New Brunswick, NJ USA

**Keywords:** T-cell acute lymphoblastic leukemia, Methylthioadenosine phosphorylase, 6-Thioguanine, Pralatrexate, Antifolate

## Abstract

**Purpose:**

To investigate the effectiveness of a combination of 6-thioguanine (6-TG) and pralatrexate (PDX) in methylthioadenosine phosphorylase (MTAP)-deficient T-cell acute lymphoblastic leukemia (T-cell ALL).

**Methods:**

CCRF-CEM (MTAP^−/−^) and Molt4 (MTAP^+/+^) T-cell ALL cell lines were treated with 6-TG or PDX and evaluated for efficacy 72 h later. NOD/SCID gamma mice bearing CEM or Molt4 xenografts were treated with 6-TG and PDX alone or in combination to evaluate antitumor effects.

**Results:**

CEM cells were more sensitive to 6-TG and PDX in vitro than Molt4. In vivo, CEM cells were very sensitive to PDX and 6-TG, whereas Molt4 cells were highly resistant to 6-TG. A well-tolerated combination of PDX and 6-TG achieved significant tumor regression in CEM xenografts.

**Conclusions:**

The loss of MTAP expression may be therapeutically exploited in T-cell ALL. The combination of 6-TG and PDX, with the inclusion of leucovorin rescue, allows for a safe and effective regimen in MTAP-deficient T-cell ALL.

## Introduction

**Fig. 1:**
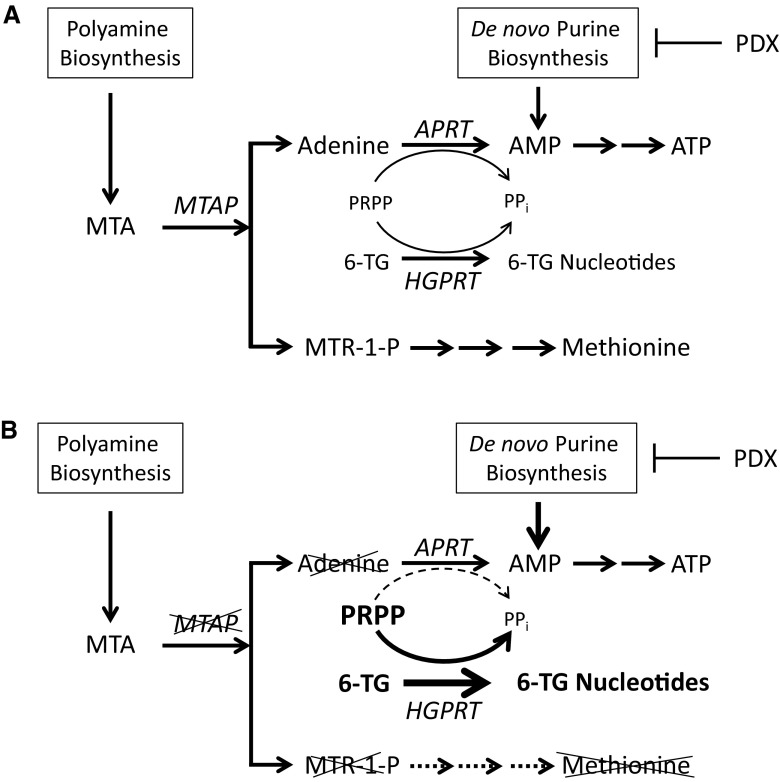
Loss of MTAP sensitizes cells to 6-thioguanine and de novo purine synthesis inhibitors. **a** MTAP allows for the salvage of adenine and methionine from methylthioadenosine (MTA) produced during polyamine synthesis. Purine analog drugs such as 6-thioguanine (6-TG) must compete with adenine for PRPP in the formation of their nucleotide derivatives, mediated by APRT for adenine and HGPRT for 6-TG. **b** In cells lacking MTAP, salvage of both methionine and adenine is prevented. These cells should become more sensitive to 6-TG as more PRPP allows greater synthesis of active 6-TG nucleotides well as de novo purine synthesis inhibitors such as pralatrexate, which inhibit the production of AMP. *6-TG* 6-thioguanine, *AMP* adenosine monophosphate, *APRT* adenosine phosphoribosyltransferase, *ATP* adenosine triphosphate, *HGPRT* hypoxanthine-guanine phosphoribosyltransferase, *PDX* pralatrexate, *PPi* pyrophosphate, *PRPP* phosphoribosyl pyrophosphate, *MTA* methylthioadenosine, *MTAP* methylthioadenosine phosphorylase. Modified from Reference [4]

The genes CDKN2A and CDKN2B (encoding the tumor suppressors p15, p16 and p19) are commonly deleted or hypermethylated in T-cell acute lymphoblastic leukemia (ALL) [1, 2]. It is common to have a co-deletion of methylthioadenosine phosphorylase (MTAP) as the MTAP gene is located adjacent to the CDKN2A/B locus [3]. Studies have shown that around 70 % of T-cell ALL cases have MTAP deletions [4, 5]. MTAP cleaves the natural by-product of polyamine synthesis, 5′-deoxy-5′-methylthioadenosine, to adenine and 5-methylthioribose-1-phosphate, which are converted to adenine nucleotides and methionine [5] (Fig. 1). Cells lacking MTAP are unable to salvage adenine through this pathway and are reliant on the *de nov*o synthesis pathway for their purine requirements. MTAP has also been implicated as a possible tumor suppressor [6]. This variation between MTAP^+/+^ cells and tumor MTAP^−/−^ cells led to several studies showing that MTAP^−/−^ cells are more sensitive to inhibitors of de novo purine synthesis [7].

Currently, the antifolate methotrexate is commonly used as a component of treatment options in pediatric and T-cell ALL in adults [8]. In contrast to the 80 % survival rate in children with T-cell ALL [9], curability of adults with this disease is poor [10]. Patients usually respond to intensive chemotherapy, but there is a high relapse rate. Allogenic transplantation may be effective, but many patients relapse or are burdened with graft-versus-host disease. Therefore, there is a need for newer and more effective treatments, both for adults and children who relapse from first-line treatment. There has been much research on the effect of MTAP expression on different leukemia and lymphoma types, and there is still much more information to uncover regarding the best drugs and optimal dosages to use when treating patients [11].

Pralatrexate (PDX) is a second generation antifolate that targets dihydrofolate reductase and inhibits de novo purine synthesis through depletion of 10-formyl tetrahydrofolate [12, 13]. PDX has a high affinity for reduced folate carrier-1 and is better retained by cells via conversion to polyglutamylates [14]. 6-thioguanine (6-TG), an antimetabolite analog of guanine, also targets de novo purine synthesis [5, 15].

In this study, we tested single-agent PDX and 6-TG as well as in combination to treat MTAP^+/+^ (Molt4) and MTAP^−/−^ (CEM) T-cell ALL cell lines and xenografts. CEM xenografts were found to be more sensitive to PDX treatment than Molt4 xenografts. CEM xenografts were also more sensitive to 6-TG, while Molt4 xenografts were resistant to 6-TG. Results of the combination of PDX, 6-TG and leucovorin against CEM xenografts show promise as an effective therapy for MTAP-deficient T-cell ALL.

## Materials and methods

### Immunoblot

Log-phase CEM and Molt4 cells were harvested by centrifugation after culture in RPMI 1640 supplemented with 10 % FBS. After brief centrifugation, cell pellets were lysed in RIPA buffer containing a commercial protease inhibitor mix (Roche, Nutley, NJ, USA). After protein quantification by the Bradford protein assay (Bio-Rad Laboratories, Hercules, CA, USA), proteins were resolved by 10 % SDS-PAGE and transferred onto a nitrocellulose membrane (Bio-Rad Laboratories). After blocking the membrane with 5 % nonfat dry milk prepared in Tris-buffered saline +0.1 % Tween-20, the membrane was incubated with the desired primary antibody according to the manufacturer’s directions at 4 °C overnight. The membrane was washed in Tris-buffered saline +0.1 % Tween-20 and incubated for 2 h at room temperature with the appropriate peroxidase-conjugated secondary antibody. Bands were visualized using an enhanced chemiluminescence kit (Pierce, Thermo Fisher Scientific, Rockford, IL, USA). Anti-MTAP (42-T), anti-α-tubulin (B-7) and anti-mouse secondary were purchased from Santa Cruz Biotechnologies (Dallas, TX, USA).

### In vitro cytotoxicity

Five-thousand Molt4 or CCRF-CEM (CEM) cells per well were plated in 96-well plates in RPMI 1640 media (Gibco) supplemented with 10 % dialyzed FBS (Invitrogen). Media containing drug was added, and plates were incubated for 72 h. The CellTiter 96 Aqueous One Solution (Promega) assay was used to assess cell viability at the end of the experiment according to the manufacturers’ protocol. Experiments were performed in triplicate. Data were analyzed using the GraphPad Prism 6 software package (GraphPad Software Inc.).

### In vivo experiments

Eight-to-ten-week-old male NOD/SCID gamma mice (a gift from Dr. Sharon Pine) were injected subcutaneously into the right flank with 15 million CEM or Molt4 cells in a 1:1 mixture of Matrigel (BD Biosciences) and PBS (Gibco). Mice were monitored until palpable tumors reached a size of 400 mm^3^. Mice were then randomized and split into treatment cohorts of eight mice as described. Drugs for study were prepared in the following manner: 6-thioguanine was dissolved in 0.15 M NaCl and heated at 70 °C. NaOH was added until a clear solution formed; the solution was sterile filtered with a 0.22-µm filter. Leucovorin was sourced from the Rutgers Cancer Institute of New Jersey’s pharmacy, and pralatrexate was provided by Spectrum Pharmaceuticals.

General toxicity was monitored through weight loss. Tumor dimensions were measured every 2–4 days using calipers, and tumor volume was calculated using the following equation: Volume = (width^2^) × (length/2). All animal experiments were approved by the Rutgers Institutional Animal Care and Use Committee.

## Results

**Fig. 2:**
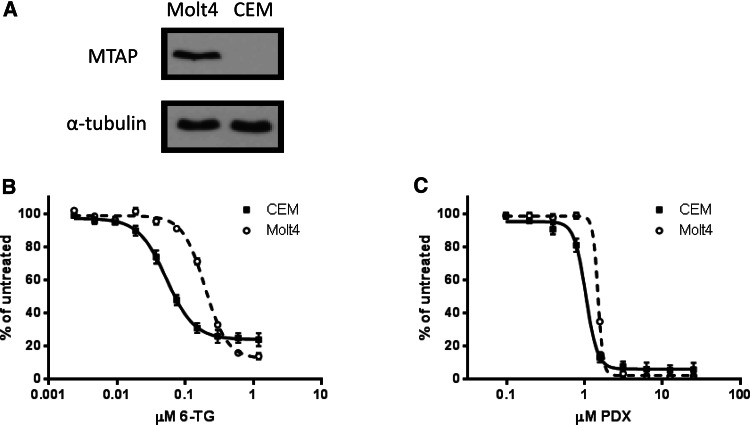
Cells lacking MTAP are more sensitive to 6-TG and pralatrexate. **a** Western blots showing the MTAP status of CEM (MTAP^−/−^) and Molt4 (MTAP^+/+^) cells. Dose response curves show percent of viable CEM or Molt4 cells remaining after 72 h of treatment with **b** 6-TG or **c** PDX. All experiments were performed in triplicate

We used two T-cell ALL cell lines to determine the effect MTAP expression has on drug sensitivity. Molt4 cells express MTAP, whereas CEM cells do not express MTAP (Fig. 2a). Both cell lines were treated with 6-TG or PDX for 72 h and assayed for viability. As expected, CEM cells were more sensitive to both 6-TG and PDX, although only 6-TG showed a sizable (~fivefold) separation between the IC_50_ for each cell line (Fig. 2b, c). This enhanced sensitivity can be attributed to the increase in 6-TG nucleotide incorporation in MTAP^−/−^ cells through the action of hypoxanthine-guanine phosphoribosyltransferase (HGPRT) [16] (Fig. 1b).

### Single-agent in vitro treatment

**Fig. 3:**
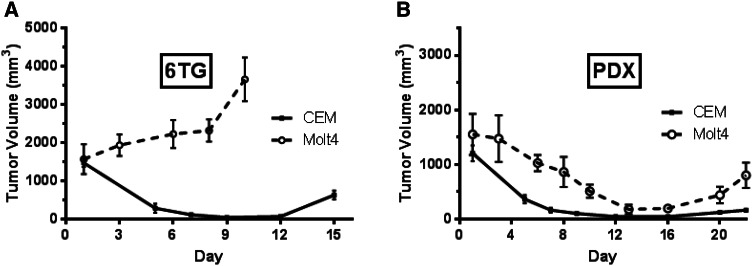
CEM xenografts are more sensitive to PDX and 6-TG than Molt4 xenografts. Mice bearing large CEM or MOLT4 xenografts were administered **a** 10 mg/kg 6-TG on days 1–5 or **b** 60 mg/kg PDX on days 1 and 5. Tumor volume was measured every few days

To evaluate the response of these drugs in vivo, NOD/SCID gamma mice bearing advanced CEM or Molt4 xenografts were treated with PDX and 6-TG. Large xenografts used in a previous experiment showed dramatic responses to PDX treatment. In response to a dosing schedule of 10 mg/kg 6-TG on days 1–5 (Fig. 3a, solid line), CEM xenografts exhibited a rapid loss in tumor burden. Molt4 xenografts demonstrated little response to 6-TG treatment (Fig. 3a, dashed line). Compared to the large response CEM xenografts had to 6-TG treatment, the lack of even moderate regression in Molt4 xenografts was notable. Treatment with 60 mg/kg PDX on days 1 and 5 resulted in tumor regression for both cell lines (Fig. 3b). Both treatments were tolerated with moderate weight loss (data not shown).

### Treatment of mice bearing xenografts of CEM cells

**Fig. 4:**
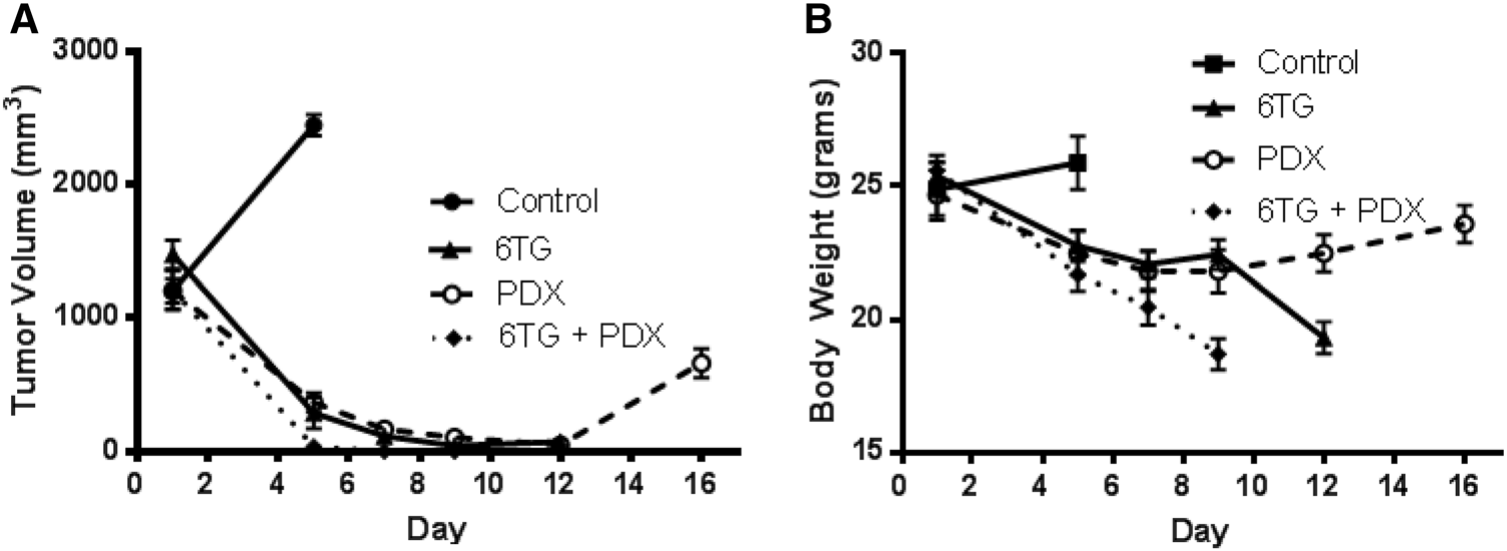
The combination of 6-TG and PDX was not tolerable, despite significant efficacy. Mice bearing advanced CEM xenografts were treated with 10 mg/kg 6-TG on days 1–8 and/or 60 mg/kg PDX on days 1 and 5. Mice treated with 6-TG alone or in combination exhibited severe weight loss and had to be killed. Xenografts on control mice reached an unacceptable size, and mice were euthanized according to the institutional protocols. Tumor volume (**a**) and bodyweight (**b**) were measured

Having established that MTAP^+/+^ xenografts have limited sensitivity to 6-TG, we focused our efforts on treating MTAP^−/−^ xenografts with a combination of 6-TG and PDX. The enhanced incorporation of 6-TG into nucleotides and lack of adenine salvage in addition to the reliance of de novo synthesis of purines in MTAP^−/−^ cells predicts potent sensitivity of these cells to 6-TG and PDX in combination. To test this, large CEM xenografts were treated using a schedule of 60 mg/kg PDX on days 1 and 5 and/or 10 mg/kg 6-TG on days 1–8. There was a complete regression of all CEM xenografts in the combination cohort (Fig. 4a). However, this combination schedule was very toxic, resulting in an unacceptable decline in bodyweight, necessitating killing of these animals (Fig. 4b). The increase in 6-TG dosing also lead to a rapid decline in bodyweight several days after the last treatment in the 6-TG cohort, indicating that a cumulative toxic dose was reached (Fig. 4b).

**Fig. 5:**
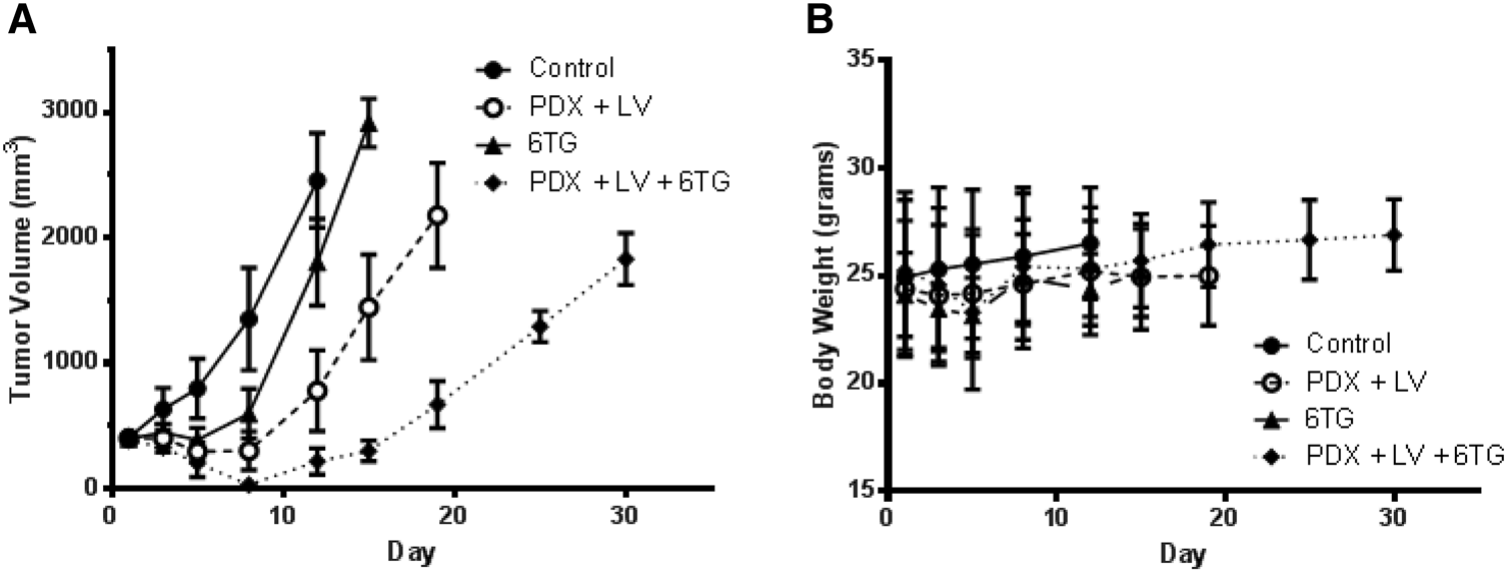
The inclusion of leucovorin to a modified 6-TG/PDX combination allows for a safe and effective dose schedule. Mice bearing 400 mm^3^ CEM xenografts were randomized into the following cohorts: untreated control; 60 mg/kg PDX on day 1 + 50 mg/kg leucovorin on days 2 and 3; 10 mg/kg 6-TG on days 1–3; 60 mg/kg PDX on day 1 + 50 mg/kg leucovorin on days 2 and 3 + 10 mg/kg 6-TG on days 1–3. Tumor volume (**a**) and bodyweight (**b**) were measured. *Asterisks*: *p* values **≤0.01; ***≤0.001; ****≤0.0001

Despite this toxicity, the complete response observed in the combination cohort was encouraging. We previously established that PDX toxicity can be abrogated using leucovorin (LV) rescue without compromising antitumor effects [17]. Therefore, we included LV into the 6-TG and PDX combination to lessen general toxicity but preserve antitumor activity. In this experiment, to demonstrate that decreasing the intensity of the regimen and treating the mice with only one cycle could result in tumor regression without toxicity, we treated cohorts consisting of 60 mg/kg PDX on day 1 + 50 mg/kg leucovorin on days 2 and 3; 10 mg/kg 6-TG on days 1–3; 60 mg/kg PDX on day 1 + 50 mg/kg leucovorin on days 2 and 3 + 10 mg/kg 6-TG on days 1–3 to better monitor the effects of the combination. Significant antitumor activity was seen in the combination cohort, with almost complete tumor regression and a prolonged recovery period (Fig. 5a), despite the administration of only one cycle. All treatment cohorts were well tolerated, and no significant loss of bodyweight occurred (Fig. 5b).

## Discussion

Although pralatexate is approved for treatment of T-cell lymphomas, studies of this drug to treat T-cell ALL have not as yet been reported. A recent study from the Children’s Oncology Group showed that high-dose MTX with LV rescue enhanced the outcome of patients with T-cell ALL [18]. Given that a high percentage of pediatric patients with T-cell ALL lack MTAP, this may be the explanation for the clinical benefit observed with MTX. Our previous study showed that PDX was even more effective than MTX in the treatment of CEM T-cell lymphoma that lacked MTAP. We also showed that subsets of peripheral T-cell lymphoma lack MTAP in a high percent of cases [4]. The study presented here provides a strong rationale for using the combination of pralatrexate and 6-thioguanine for the treatment of leukemias and lymphomas lacking MTAP [5]. Based on these data, a clinical study using this combination strategy has been initiated in T-cell ALL.

## References

[1] Drexler HG (1998). Review of alterations of the cyclin-dependent kinase inhibitor INK4 family genes p15, p16, p18 and p19 in human leukemia–lymphoma cells. Leukemia.

[2] Maloney KW, McGavran L, Odom LF, Hunger SP (1999). Acquisition of p16INK4A andp15INK4B gene abnormalities between initial diagnosis and relapse in children with acute lymphoblastic leukemia. Blood.

[3] Hori Y, Hori H, Yamada Y (1998). The methylthioadenosine phosphorylase gene is frequently co-deleted with the p16INK4a gene in acute type adult T-cell leukemia. Int J Cancer J Int Cancer.

[4] Bertino JR, Waud WR, Parker WB, Lubin M (2011). Targeting tumors that lack methylthioadenosine phosphorylase (MTAP) activity. Cancer Biol Ther.

[5] Munshi PN, Lubin M, Bertino JR (2014). 6-thioguanine: a drug with unrealized potential for cancer therapy. Oncologist.

[6] Kadariya Y, Yin B, Tang B (2009). Mice heterozygous for germ-line mutations in methylthioadenosine phosphorylase (MTAP) die prematurely of T-cell lymphoma. Cancer Res.

[7] Chen ZH, Olopade OI, Savarese TM (1997). Expression of methylthioadenosine phosphorylase cDNA in p16-, MTAP- malignant cells: restoration of methylthioadenosine phosphorylase-dependent salvage pathways and alterations of sensitivity to inhibitors of purine de novo synthesis. Mol Pharmacol.

[8] Pui C-H, Jeha S (2007). New therapeutic strategies for the treatment of acute lymphoblastic leukaemia. Nat Rev Drug Discov.

[9] Roti G, Stegmaier K (2014). New approaches to target T-ALL. Front Oncol.

[10] Kozlowski P, Åström M, Ahlberg L (2014). High relapse rate of T-cell acute lymphoblastic leukemia in adults treated with Hyper-CVAD chemotherapy in Sweden. Eur J Haematol.

[11] Efferth T, Miyachi H, Drexler HG, Gebhart E (2002). Methylthioadenosine phosphorylase as target for chemoselective treatment of T-cell acute lymphoblastic leukemic cells. Blood Cells Mol Dis.

[12] Sirotnak FM, DeGraw JI, Colwell WT, Piper JR (1998). A new analogue of 10-deazaaminopterin with markedly enhanced curative effects against human tumor xenografts in mice. Cancer Chemother Pharmacol.

[13] Visentin M, Unal ES, Zhao R, Goldman ID (2013). The membrane transport and polyglutamation of pralatrexate: a new-generation dihydrofolate reductase inhibitor. Cancer Chemother Pharmacol.

[14] Izbicka E, Diaz A, Streeper R (2009). Distinct mechanistic activity profile of pralatrexate in comparison to other antifolates in in vitro and in vivo models of human cancers. Cancer Chemother Pharmacol.

[15] Tang B, Testa JR, Kruger WD (2012). Increasing the therapeutic index of 5-fluorouracil and 6-thioguanine by targeting loss of MTAP in tumor cells. Cancer Biol Ther.

[16] Nelson JA, Carpenter JW, Rose LM, Adamson DJ (1975). Mechanisms of action of 6-thioguanine, 6-mercaptopurine, and 8-azaguanine. Cancer Res.

[17] Tedeschi PM, Kathari YK, Farooqi IN, Bertino JR (2014). Leucovorin rescue allows effective high-dose pralatrexate treatment and an increase in therapeutic index in mesothelioma xenografts. Cancer Chemother Pharmacol.

[18] Asselin BL, Devidas M, Wang C (2011). Effectiveness of high-dose methotrexate in T-cell lymphoblastic leukemia and advanced-stage lymphoblastic lymphoma: a randomized study by the Children’s Oncology Group (POG 9404). Blood.

